# Isoflurane-lipid emulsion injection as an anticonvulsant and neuroprotectant treatment for nerve agent exposure

**DOI:** 10.3389/fphar.2024.1466351

**Published:** 2024-10-02

**Authors:** Jishnu K. S. Krishnan, John R. Moffett, Narayanan Puthillathu, Erik A. Johnson, Aryan M. Namboodiri

**Affiliations:** ^1^ Uniformed Services University of the Health Sciences, Anatomy, Physiology and Genetics Department and Neuroscience Program, Bethesda, MD, United States; ^2^ Department of Chemistry and Biochemistry, Interdisciplinary Graduate Program in Biophysics, Ohio State University, Columbus, OH, United States; ^3^ Department of Neuroscience, United States Army Medical Research Institute of Chemical Defense, Gunpowder, MD, United States

**Keywords:** organophosphate poisoning, paraoxon, drug repurposing, intravenous drug administration, convulsant antidote for nerve agents

## Abstract

We have shown that briefly inhaled isoflurane rapidly halts convulsions and protects the central nervous system (CNS) from organophosphate-induced neuronal loss when administered at 5% for 5 min, even as late as 1 h after organophosphate exposure. In the current study we investigated if an injectable form of isoflurane was as effective as inhaled isoflurane. We used a mixture of 10% isoflurane dissolved in an IV-compatible lipid-water emulsion for intravenous administration. Rats with an implanted jugular vein cannula were infused with 1,000 μL of the 10% isoflurane-lipid emulsion (ILE) mixture at a rate of 200 μL per minute, which achieved full anesthesia lasting approximately 10 min. When administered 30 min after a highly lethal dose of the organophosphate insecticide paraoxon (POX), the short-duration administration halted convulsions over the course of the study and prevented the great majority of neuronal loss as shown by Fluoro-Jade B staining (FJB). Our results indicate that injectable isoflurane is very effective for treating organophosphate poisoning, negating the need for vaporizer equipment and enabling intravenous therapy.

## 1 Introduction

Isoflurane is an inhaled, volatile anesthetic which is administered using a vaporizer, and this equipment is widely available at medical treatment facilities. We have repurposed isoflurane, an FDA-approved anesthetic, as an anti-convulsant and neuroprotective drug for use in the treatment of organophosphate poisoning. Brief, high-dose administration of isoflurane is the opposite of its use as a surgical anesthetic, where low doses are administered for prolonged periods of time and is a novel application for seizure control and neuroprotection against organophosphate toxicity. The primary drawback of using this method to treat organophosphate poisoning is that it requires patients to be transported to medical treatment facilities where anesthesia equipment and trained personnel are available. The development of an intravenous isoflurane-based treatment would provide greater treatment flexibility by allowing administration under conditions where vaporizers are unavailable.

We have shown that isoflurane can be used as a single-administration drug to treat organophosphate poisoning, in addition to its current role as a prolonged-administration surgical anesthetic. Using very brief administration times of only 5 min, POX-induced convulsions are stopped without the need for subsequent administrations (Krishnan et al., 2017). This treatment also prevents brain edema and the great majority of neuronal loss, even when treatment is delayed up to 1 h after POX administration (Puthillathu et al., 2023). We hypothesized that an intravenous delivery method could be developed for the use of isoflurane to treat organophosphate poisoning. We tested this hypothesis and found that an ILE containing 10% isoflurane could be used to stop convulsions when given to ∼300 gm rats at a flow rate of 200 μL/min for 5 min (1 mL of emulsion total). These findings provide proof-of-concept that isoflurane emulsions can be used as an intravenous treatment for organophosphate poisoning.

The relevance of these studies to organophosphate poisoning is that the effectiveness of the current treatment regimen of oxime, atropine sulfate and benzodiazepine is time-dependent and may not fully control convulsions and seizures or sufficiently protect the brain from neuronal loss. Our treatment method addresses these shortcomings. However, the administration of isoflurane via anesthesia vaporizer equipment requires that patients be transported to medical treatment facilities that are equipped for surgical care. It would therefore be advantageous to develop a treatment that can be given intravenously and thus negate the need for vaporizers. The current study provides proof-of-principle that this can be achieved with intravenous administration of an ILE.

## 2 Methods

Animal experiments were conducted following guidelines from the NIH for the care and use of laboratory animals, and the protocols were approved by the animal care and use committee of the Uniformed Services University of the Health Sciences, Bethesda, MD. Eight-week-old adult male Sprague-Dawley rats, (260 ± 50 g) implanted with jugular vein cannulas were acquired from Envigo RMS, (Indianapolis, IN). Animals were housed in individual cages in environmentally controlled rooms (20°C–23°C, ∼44% humidity, 12 h light/dark cycle, 350–400 lx, lights on at 6:00 a.m.), with food (Teklad Global; 18% protein #2018 rodent diet; Harlan Laboratories, IN) and water available continuously. Rats fitted with a jugular cannula were allowed to recover after surgery for 2 weeks before the experiments. Animal handling was minimized to reduce stress. Reagents were from Sigma Aldrich (St. Louis, MO).

We tested ILE given intravenously by jugular cannulas in rats using a POX model of organophosphate poisoning (Puthillathu et al., 2023). The ILE was prepared fresh by adding isoflurane (10% by volume) to an IV-compatible lipid emulsion, Intralipid-30 (a sterile Intravenous lipid-water emulsion containing 30% lipid) and mixing for 20 min at 200 rpm on an orbital shaker in a tightly capped glass bottle with Teflon lid. The solution was kept tightly capped and cold until use to prevent any loss of isoflurane due to vaporization. Tests were done to determine the proper dosing for rats weighing between 260 and 320 g.

We then tested the effectiveness of the isoflurane emulsions in rats given a highly lethal dose of POX (4 mg/kg; ∼9x LD_50_). For these experiments, POX solutions were freshly prepared by adding 10 µL of stock solution (POX ethyl; 1.27 g/mL) to 3 mL of ice-cold phosphate buffered saline (PBS) and mixing thoroughly. POX (4 mg/kg) (Deshpande et al., 2014; Misik et al., 2015) was administered subcutaneously, followed immediately by intramuscular atropine sulfate (2 mg/kg) and intramuscular pralidoxime (2-PAM, 25 mg/kg). Due to the high dose of POX used, immediate administration of atropine and 2-PAM is required for a sufficient number of animals to survive for 24 h and complete the experiment. Control rats were implanted with cannulas but were not treated with 2-PAM, atropine sulfate or isoflurane. The ILE emulsions were administered by a syringe infusion pump connected to the jugular cannula. The flow rate was set at 200 µL per minute which was found to be optimal for rats weighing 260-320 gm. The ILE was administered for 5 min starting 30 min after POX administration (1 mL total administered). The animal groups included exposed (POX, n = 7), treated (POX followed by ILE 30 min later, n = 8) and control (cannula implantation, but no other treatment, n = 5).

Animals were monitored for convulsions as previously reported (Krishnan et al., 2017; Puthillathu et al., 2023). Rats were observed independently by two trained researchers immediately after POX administration for convulsion severity. Monitoring was done to ensure that all experimental animals reached a sustained convulsion level (stage 5 or 6) for 10 min or longer.

To determine neuronal loss in response to POX poisoning, and the prevention of neuronal loss by treatment with ILE, we assessed Fluoro-Jade B (FJB) staining in the animals 24 h after POX administration. This is the time point where maximal neuronal degeneration can be detected by this method. Twenty-4 hours after POX administration, the rats were anesthetized with a lethal dose of Euthasol (1 mL) and perfused transcardially with 300 mL of 4% freshly de-polymerized paraformaldehyde prepared in PBS (pH adjusted to 7.4). Brains were carefully extracted and fixed overnight in the same fixative, after which they were cryoprotected in a 10%, 20%, and 30% sucrose solution series prepared in PBS. Tissue sectioning and FJB staining were done by FD Neurotechnologies (Columbia, MD). Brains were sectioned by cryostat at a thickness of 40 μm, and 20 tissue sections were collected from each animal in the region from −1.8 to −3.5 mm from Bregma. A complete digital representation of each tissue section was acquired with a ×10 objective using a Zeiss Axioscan Z1 automated slide scanner. These images were used for neuropathology analyses.

Neuropathology scoring was done by examining each region in all tissue section images from each animal in the study. Twenty tissue sections were examined from each animal. The areas of interest included the central thalamus, dorsal thalamus, neocortex, all regions of the amygdala, the hippocampus, the piriform cortex and the endopiriform area. Neuropathology severity scoring was assigned in 11 ranks ranging from 0–10, representing no FJB stained cells for a score of 0 and maximal neuronal staining (most or all neurons in the region being stained) giving a score of 10 (see Supplementary Material for images showing the range of FJB staining). Ranks were assigned in approximate 10% intervals.

## 3 Results

### 3.1 ILE administration

We tested several flow rates for the delivery of the 10% ILE and found that for 260–320 gm rats, an infusion rate of 200 μL/min for 5 min produced sustained unconsciousness within 40–60 s, and animals remained unconscious for 8–10 min. Once the ILE-treated animals awakened, they resumed normal activity within approximately 10 min. Two out of 17 animals given the ILE failed to become unconscious, presumably due to a malfunction with their jugular cannulas, and were removed from the study. Fifteen rats in the experimental groups (8 exposed to POX only, 7 exposed to POX and given the ILE) were included in the study, along with 5 naïve control rats that were not exposed to POX or treated with ILE.

### 3.2 Racine behavioral monitoring

Within two to 3 minutes of POX administration, all rats displayed convulsions at levels 5 to 6 on the Racine scale. Convulsions continued at a level of 3 through 5 for the next 30 min. ILE was infused by jugular cannula starting 30 min after POX treatment. Convulsions in the ILE-treated group were halted as soon as the animals became unconscious, which occurred within 1 minute after the start of the infusion. After 5 min, the ILE infusion was stopped and the cannula was disconnected from the infusion pump. The ILE-treated animals regained consciousness after another 4–6 min. After awakening, the treated animals did not show any signs of convulsions and exhibited very low activity except for occasional positional adjustments for the next hour or longer. This low-activity period was not observed in animals given only the ILE, who showed normal activity levels within 8–10 min of regaining consciousness. In contrast, all animals that were given POX but not treated with ILE continued to exhibit convulsions at a Racine scale of 3–5 for the remainder of the observation period (4 h).

Overall mortality was 35% (9 out of 26). These animals died within 5–20 min of POX administration, before isoflurane was administered. At the 24-h time point, all of the rats treated with the ILE were energetic and appeared normal. In contrast, the animals that were not treated with the ILE were very lethargic.

### 3.3 Neuropathology

**FIGURE 1:**
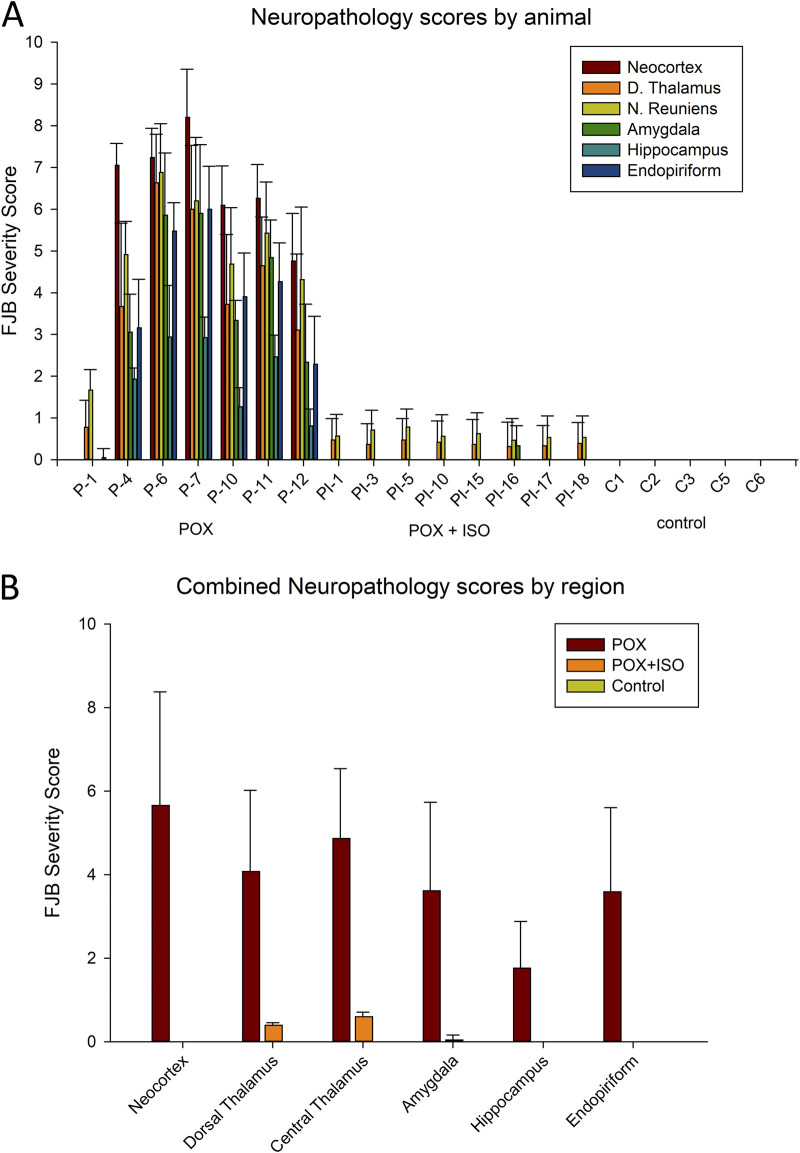
Neuropathology as determined by FJB-staining. Animal groups included injured (POX = paraoxon, n = 7), treated (paraoxon plus ILE given 30 min later, n = 8) and naïve control (cannula implantation, but no other treatment, n = 5). Panel **(A)**: FJB severity scores in each animal in the study. Error bars show the standard deviation in each brain area among the tissue sections for each animal. Results for each animal are based on observations in 14–20 tissue sections (some areas, including the nucleus reuniens, are only visible in a limited rostro-caudal extent and so fewer tissue sections contribute to the neuropathology score). Animal identification numbers and experimental groups are shown on the X-axis, and FJB scores (from 0–10) are given on the Y-axis. In the ILE-treated rats, very minor FJB staining was observed in the nucleus reuniens, dorsal thalamus and amygdala, but not in the neocortex, hippocampus or piriform/endopiriform area. Panel **(B)**: FJB severity scores by region in the 3 experimental groups. Neuronal FJB staining was most extensive in neocortex and central thalamus, and was lowest in the hippocampus. Small numbers of FJB stained neurons in the ILE-treated group were only observed in the dorsal and central thalamus and the amygdala, but no neuronal FJB staining was observed in the other 3 regions. Error bars in panel B indicate standard deviation for the inter-animal FJB-staining variability in each brain region. One-way ANOVA on ranks indicated all POX + ISO neuropathology scores were significantly different from the scores in untreated animals (P < 0.002).

**FIGURE 2:**
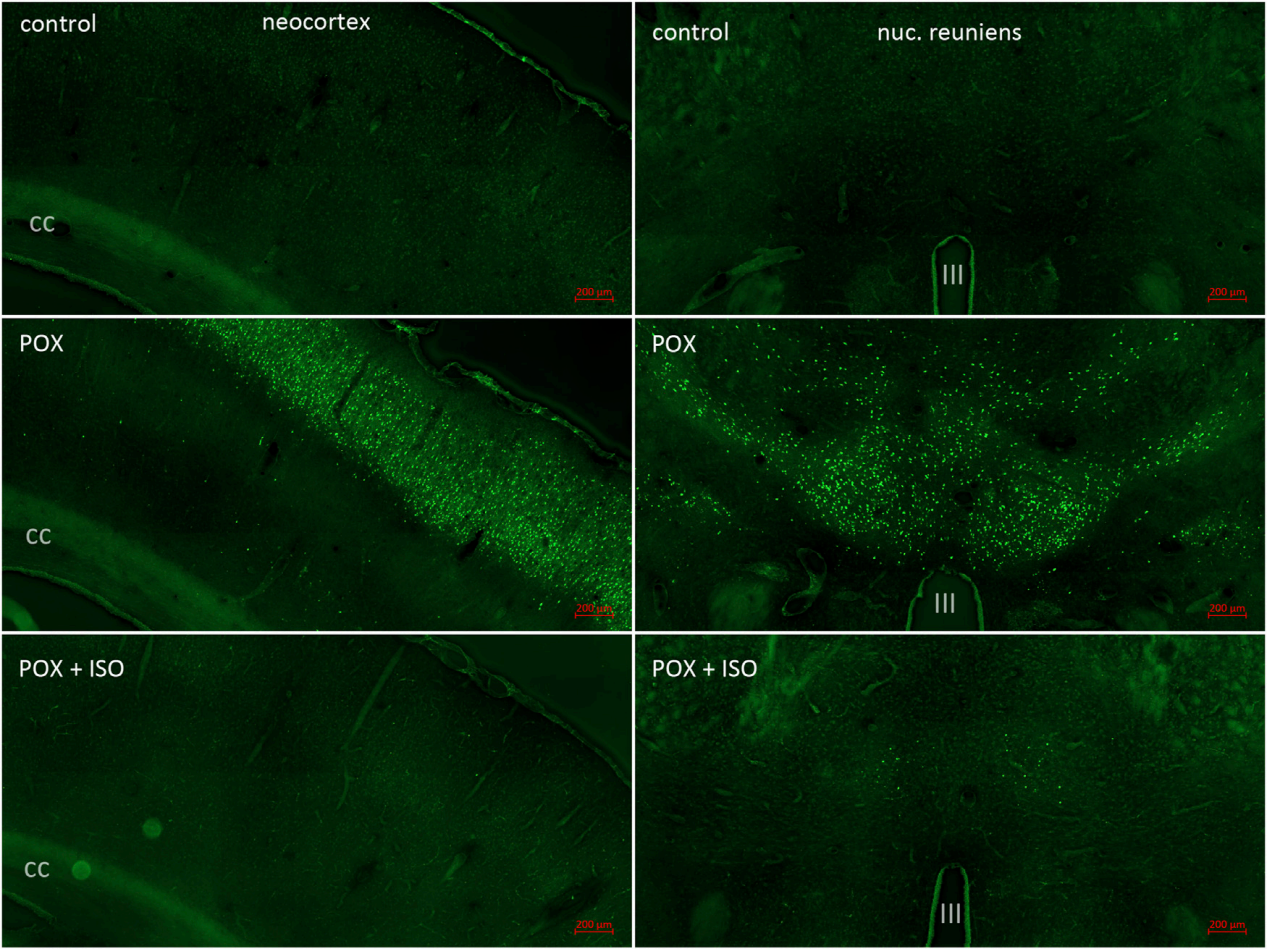
FJB staining in neocortex and the central thalamus including the nucleus reuniens. Neocortex was a major site of POX-induced neuronal damage as shown by FJB staining (left panels; cc = corpus callosum). FJB staining in cortex was variable in the animals not treated with ILE, ranging from no FJB staining in one animal to very extensive neuronal damage in the other 6 animals (see Figure 1A). No FJB staining was observed in the neocortex of any of the animals treated with the ILE (see Figure 1B). In the central thalamus, including the nucleus reuniens, FJB staining was moderate to strong in all of the animals not treated with the ILE (right panels; III = third ventricle). Very low FJB staining was observed in the nucleus reuniens from all of the rats treated with the ILE (bottom right panel).

FJB staining was used to determine neuronal damage 24 h after POX administration. FJB staining in the untreated rats was extensive in many telencephalic and diencephalic regions. Neuronal injury was extensive in most of the POX-injured animals that did not receive ILE treatment (Figure 1). Brain regions in which substantial FJB staining was observed included the neocortex, dorsal and central thalamus, the amygdala, piriform cortex and the hippocampus. Out of 7 rats that were administered POX, but not treated with the ILE, one rat had very minimal neuronal damage (rat ID: P-1), whereas the other 6 had extensive neuronal damage (Figure 1A). In our rat model of organophosphate poisoning using high-dose POX, the two regions with the greatest neuronal damage were neocortex and central thalamus, including the nucleus reuniens (Figure 2). Additional regions with significant neuronal damage included the dorsal thalamus, the amygdala, the hippocampus and the piriform/endopiriform area.

**FIGURE 3:**
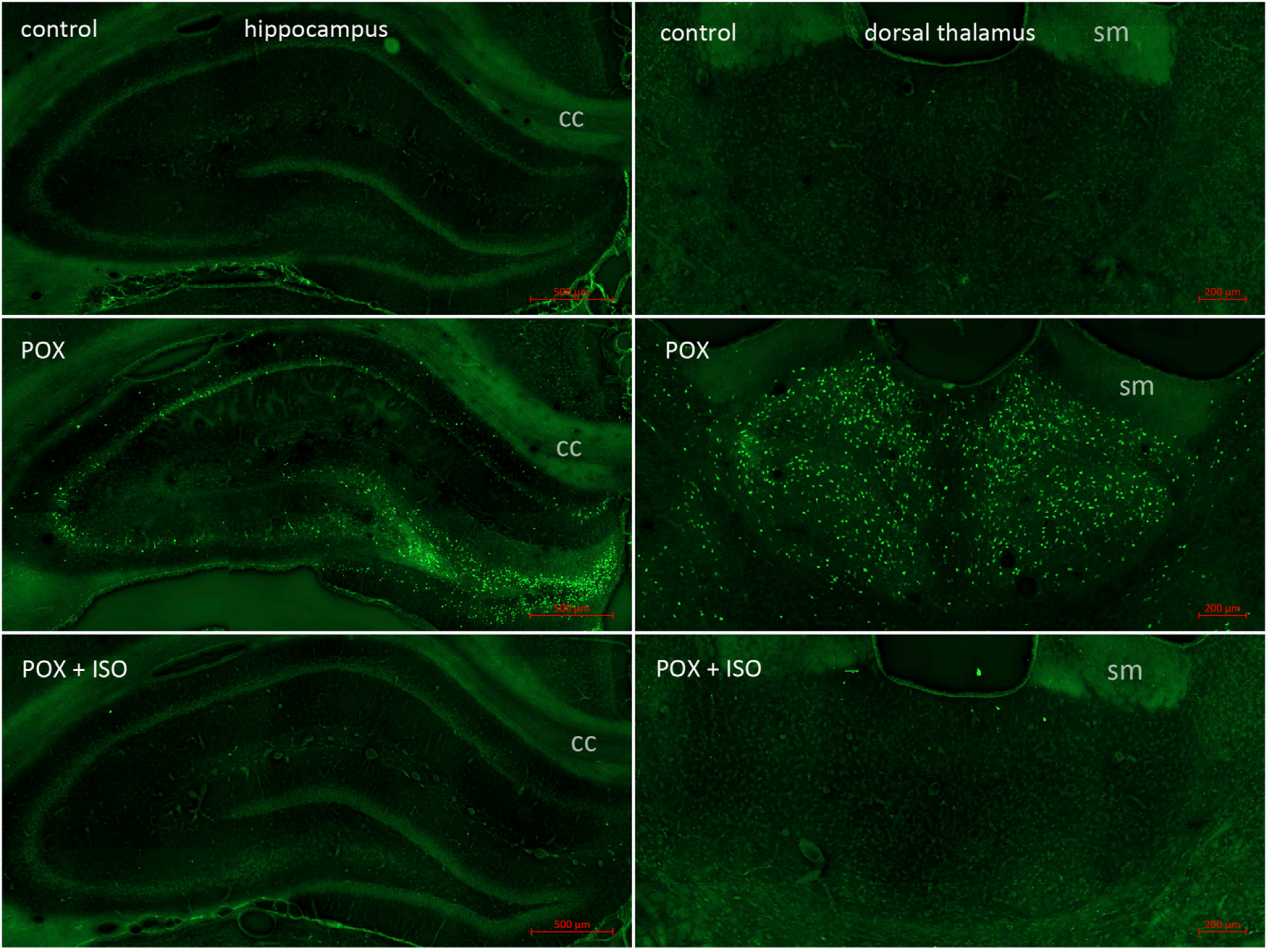
FJB staining in the hippocampus and dorsal thalamus. In our POX model, neuronal FJB staining was relatively less extensive in the hippocampus than in regions such as neocortex and central thalamus (left panels; also see Figure 1B; cc = corpus callosum). Neuronal FJB staining was observed in pyramidal cells in CA1, CA2 and CA3, as well as in neurons in the polymorph layer. No neuronal FJB staining was observed in the hippocampus of any of the ILE-treated animals. The dorsal thalamus was another site of extensive neuronal FJB staining in the animals given POX but not treated with the ILE (center right panel; sm = stria medularis). Very minimal neuronal FJB staining was observed in the dorsal thalamus of all of the ILE-treated animals (bottom right panel, also see Figure 1A).

**FIGURE 4:**
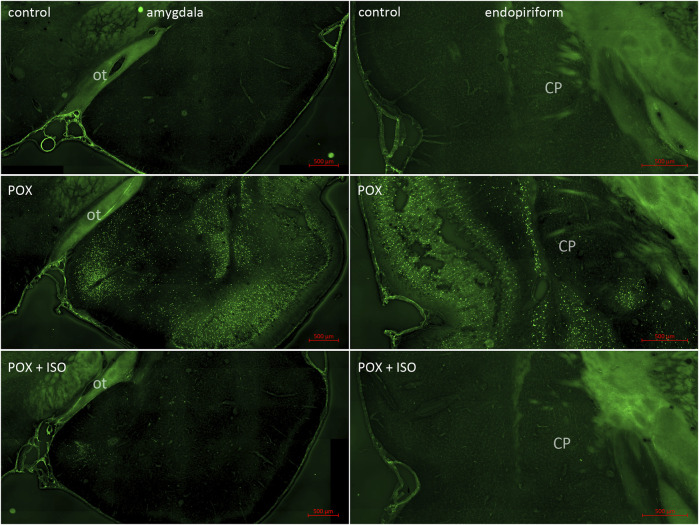
FJB staining in the amygdala complex and piriform cortex/endopiriform area. The amygdala was one of the regions with moderate to strong neuronal FJB-staining (left panels; ot = optic tract). Only one out of 8 of the ILE-treated animals had residual neuronal FJB staining, whereas no stained neurons were observed in the other 7 animals (see Figure 1A). Neuronal FJB staining was moderate to strong in the POX group (center right panel; CP = caudate/putamen), but no neuronal FJB staining was observed in the POX + ISO group (bottom right panel).

In the neocortex of the injured animals (somatosensory cortex, retrosplenial cortex, visceral cortex and agranular cortex), most FJB stained neurons were observed in layers II and III, with a relatively small number of stained cells occurring throughout deeper layers (Figure 2). The severity scores ranged from 5 to 8 in the neocortex, making this the region with the most extensive damage (Figure 1). Interestingly, no FJB staining was observed in the neocortex of any of the ILE-treated animals, indicating that neuroprotection was greatest in this region (Figure 1). In the central thalamus, the major sites of neuronal damage included the entire nucleus reuniens and portions of the ventromedial nucleus, the submedial nucleus and zona incerta (Figure 2). The nucleus reuniens was the region with the most residual FJB-staining in the animals treated with the ILE, but the average severity score was low (0.6; Figure 1B). In the hippocampus FJB-stained neurons were observed in CA1, CA2, and CA3 pyramidal cells, and cells in the polymorph layer (Figure 3). In the dorsal thalamus, FJB staining was observed in neurons in the paraventricular nucleus, the anterodorsal, lateral dorsal and medial dorsal thalamic nuclei (Figure 3). FJB-stained neurons were observed in all portions of the amygdala (Figure 4), a region with modest damage on average, ranging from 1 to 3 on the severity scale (Figure 1A). The piriform cortex and endopiriform area showed moderate FJB-severity scores (Figure 4) in the animals that did not receive the ILE, ranging from 2 to 6. No FJB-staining was observed in the amygdala or hippocampus in any of the ILE-treated animals (Figure 1).

## 4 Discussion

In this study, we have demonstrated the proof-of-principle that ILE can be given by intravenous injection (10% isoflurane by volume), and that this brief 5-min treatment blocks POX-induced convulsions rapidly without the need for re-administration. This single-dose ILE treatment also prevented the vast majority of neuronal damage when administered 30 min after POX. The currently accepted treatment regimen for organophosphate poisoning includes an oxime such as 2-PAM plus atropine sulfate, typically administered intramuscularly by use of an auto-injector. If convulsions are present, then benzodiazepines such as diazepam or midazolam are given. All 3 of these treatments are often given repeatedly as their effectiveness wanes. In particular, benzodiazepines can lose effectiveness within 30–40 min of nerve agent exposure and repeated administration can have diminishing results due to the saturation of the GABA_A_ receptor system. Our treatment provides an adjunct anti-convulsant treatment that halts convulsions rapidly and does not have to be re-administered because the convulsions do not return as they can in the case of benzodiazepines. Further, we confirm that isoflurane has neuroprotective properties in the treatment of organophosphate poisoning that benzodiazepines lack (Krishnan et al., 2017; Puthillathu et al., 2023; Swissa et al., 2020). The current findings further validate the use of single-dose isoflurane treatment for OP poisoning and verify that longer duration isoflurane administration is not required for the anti-convulsant or neuroprotective effects.

ILEs have proven safe and effective for anesthesia in humans (Huang et al., 2014; Li et al., 2014; Yang et al., 2021). In clinical trials, loss of consciousness (LOC) was observed in all patients given an ILE dose containing 22.6 mg/kg of isoflurane or higher (Huang et al., 2014). The onset of LOC typically occurred about 40 s after the initiation of the infusion, which was consistent with our observations in rats. A single bolus injection of 4 mL of ILE with a dose of 36.8 mg/kg delivered over 10 s resulted in LOC that lasted an average of 7 min (Huang et al., 2014). Studies on the solubility of isoflurane in intralipid emulsions found that the maximal concentration for intralipid-30 that would avoid separation was about 8.5% (Zhou et al., 2006). We used a somewhat higher concentration of 10% but used extensive mixing in glass vials (20 min at 200rpm on a rotary shaker) and also kept the vials tightly capped and on wet ice until use to maintain the isoflurane concentration.

Isoflurane has been shown previously, by Bar-Klein and colleagues, to prevent blood-brain-barrier disruption, neuronal damage, astrogliosis, as well as the development of post-injury epilepsy in a rat model of POX poisoning (Bar-Klein et al., 2016). In their study, they administered much lower doses of POX than the current study and used repeated 1-h administrations of lower-dose isoflurane (1% to 2% delivered in 100% oxygen). We have found that high-dose isoflurane can be administered for only 5 min to achieve similar results. The brief, 5-min IV administration of ILE in the current study was intended to replicate our findings with 5 min inhalation administration of isoflurane in the same POX rat model (Puthillathu et al., 2023). The 5-min administration proved fully effective at blocking convulsions until the end of the study (24 h), as well as dramatically reducing neuronal injury as shown by FJB staining, using both administration routes. It is unclear how such brief administration times act to block convulsions, without the need for re-administration, but previous studies have found substantial changes in gene expression using short-duration isoflurane administration. Delivery of 2% isoflurane in oxygen for 15 min was shown to significantly alter gene expression in rats (Lowes et al., 2017). Gene ontology analysis from tissue collected 6 h after isoflurane administration showed that 23 gene expression changes were associated with the anesthesia, and of these, 12 genes were associated with neurotransmitter transport and release. These results indicate that isoflurane has lasting effects on gene expression that do not require prolonged administration times. Currently, we are examining the proteomic changes in the brain in response to short-duration isoflurane exposure in the POX model of OP poisoning. The repurposing of isoflurane as a single-dose drug, rather than a long-duration anesthetic, warrants further investigation in organophosphate poisoning, as well as in other neuroprotective and cardioprotective treatments (Wu et al., 2017; Zhang et al., 2016; Zhang et al., 2017).

## Data Availability

The raw data supporting the conclusions of this article will be made available by the authors, without undue reservation.
