# Erratum: Isoflurane-lipid emulsion injection as an anticonvulsant and neuroprotectant treatment for nerve agent exposure

**DOI:** 10.3389/fphar.2024.1512783

**Published:** 2024-10-28

**Authors:** Frontiers Production Office

**Affiliations:** Frontiers Media SA, Lausanne, Switzerland

**Keywords:** organophosphate poisoning, paraoxon, drug repurposing, intravenous drug administration, convulsant antidote for nerve agents

Due to a production error, the Supplementary Materials of the published article were erroneously omitted. The Supplementary figures are now published, and the section **Supplementary Material** has been added to the published article.

The publisher apologizes for this error. The original article has been updated.

