# Supplementary material for: Isoflurane-lipid emulsion injection as an anticonvulsant and neuroprotectant treatment for nerve agent exposure
Source: Front Pharmacol. 2024 Oct 2;15:1466351. doi: 10.3389/fphar.2024.1466351 (PMC11479933; doi:10.3389/fphar.2024.1466351)
Supplement: Supplementary file 1 [file DataSheet1.docx]

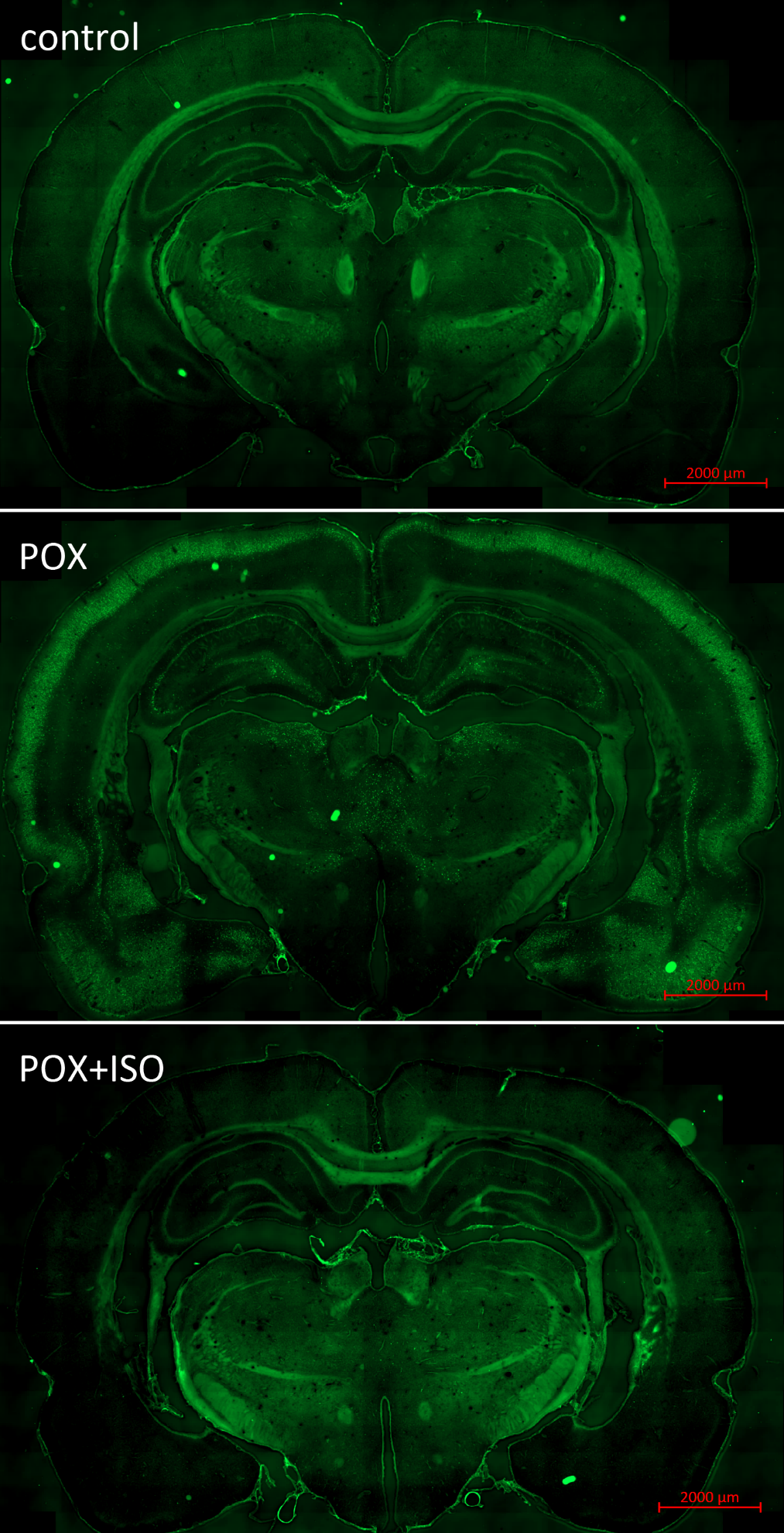


**Supplementary Figure 1: Neuronal injury as shown by FJB staining in the whole brain.** In naïve control animals, no FJB-stained neurons were observed. Staining in the control animals was limited to non-specific staining of fiber pathways. In animals receiving POX but not given the ILE, neuronal FJB staining was moderate to severe in many brain regions including the neocortex, hippocampus, central and dorsal thalamus, hippocampus and the amygdala. In contrast, only very minor neuronal FJB staining was observed in several regions of the ILE-treated rats, including the central and dorsal thalamus in all animals, as well as in the amygdala in one out of 7 animals.


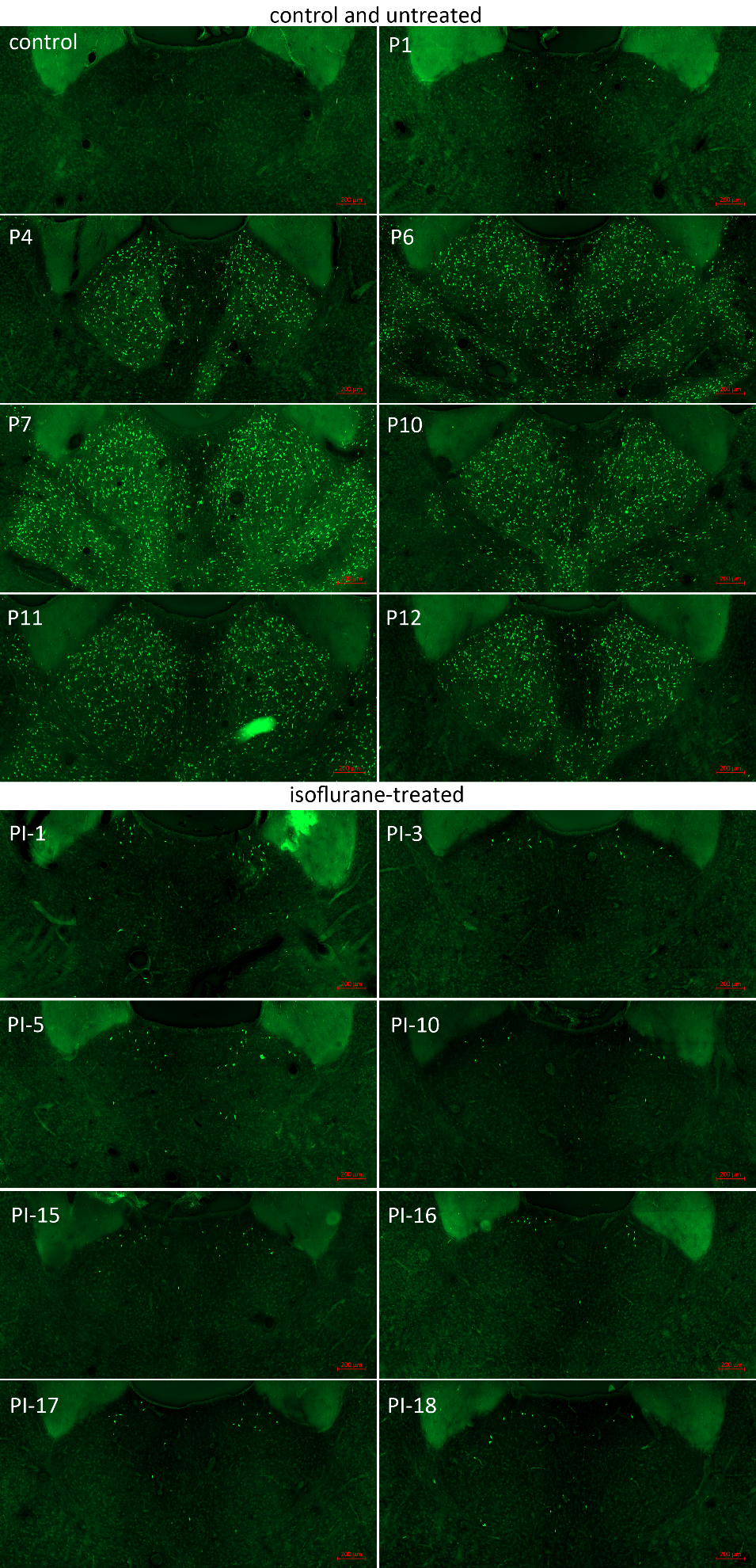


**Supplementary Figure 2: Neuronal injury as shown by FJB staining in the dorsal thalamus.** Images of the dorsal thalamus at the same rostro-caudal level are shown for all animals included in the study. The dorsal thalamus was one of the regions where severe neuronal damage was observed in the group exposed to POX, but not treated with ILE, and where minor FJB-staining was observed in all of the ILE-treated rats.


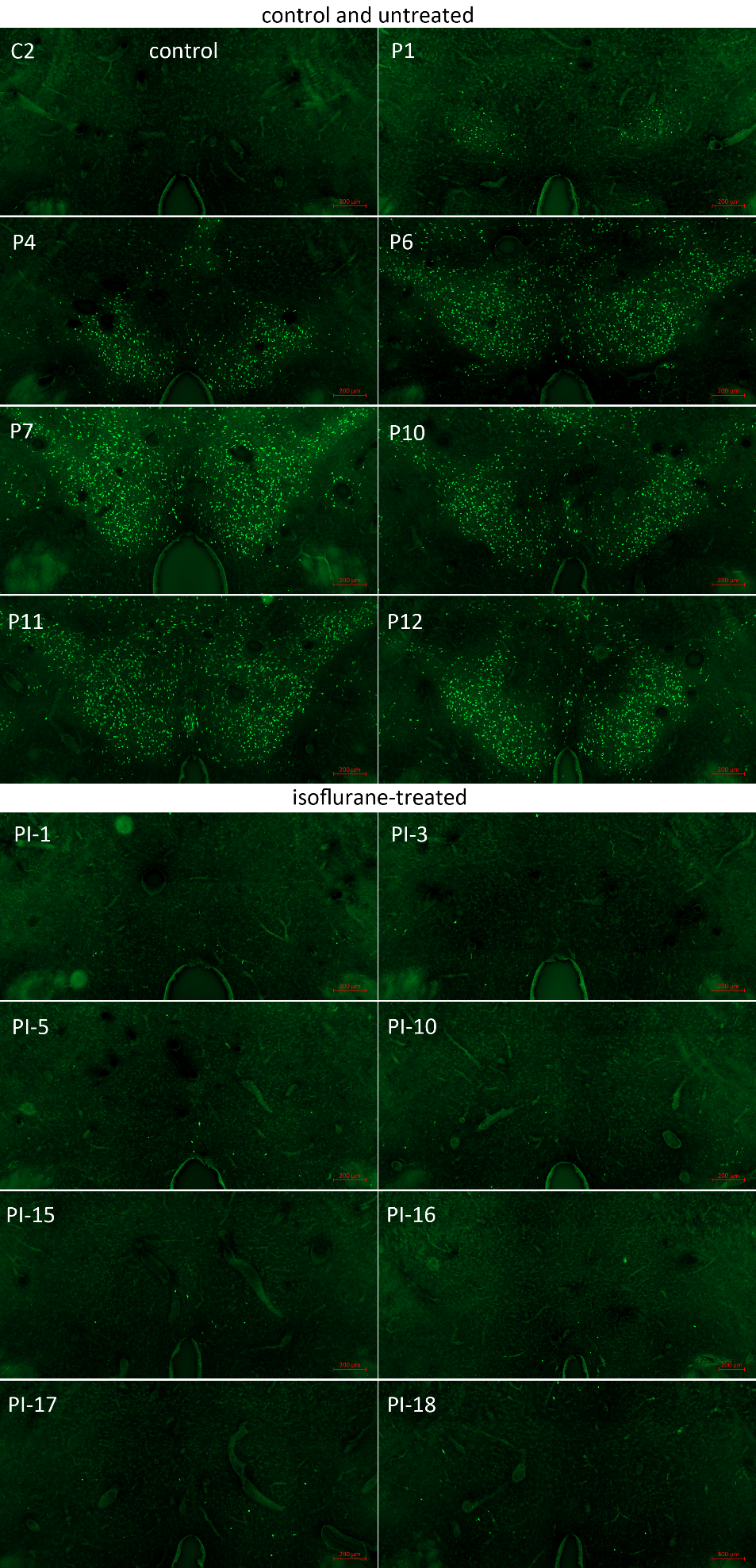


**Supplementary Figure 3: Neuronal injury as shown by FJB staining in the central thalamus, including the nucleus reuniens.** Images of the central thalamus at the same rostro-caudal level are shown for all animals included in the study. The central thalamus was one of the regions where severe neuronal damage was observed in the group exposed to POX, but not treated with ILE. In contrast, in the ILE-treated animals only very minor FJB-staining was observed.
